# Social norm intervention in real‐world supermarkets in the Netherlands: A quasi‐experimental evaluation of effects on perceived social norms and meat substitute purchases

**DOI:** 10.1111/aphw.70159

**Published:** 2026-05-08

**Authors:** Sofia M. M. Wolfswinkel, Sanne Raghoebar, Emely de Vet, Maartje P. Poelman

**Affiliations:** ^1^ Consumption & Healthy Lifestyles Group Wageningen University & Research Wageningen The Netherlands; ^2^ Urban Economics Wageningen University & Research Wageningen The Netherlands

**Keywords:** dynamic norms, intervention, meat, meat substitutes, real‐world supermarkets, social norms

## Abstract

Social norm perceptions are implicit rules of conduct that describe what is normal and acceptable behavior. Changing social norm perceptions through textual and physical social norm communications can be a promising approach to changing behavior. Past studies, however, primarily relied on controlled (lab) experiments, leaving it unanswered to what extent these norm communications relate to social norm *perceptions* in a complex context. The present study addresses this issue for meat substitute purchases: to what extent can a social norm intervention in real‐world supermarkets stimulate meat substitute purchases, and to what extent do social norm perceptions favoring meat substitute purchases mediate this relationship? A 13‐week social norm intervention was implemented in three experimental supermarkets (with *n* = 3 matched control stores). Social norms were communicated textually on stickers and banners in the meat aisle and physically through increased shelf space for meat substitutes and island shelves with legumes. A total of *N* = 639 participants (*n* = 327 control) completed a survey upon exiting the supermarket, measuring social norm perceptions and collecting receipts to measure meat substitute purchases in grams. Results show that the likelihood of meat substitute purchases was *OR* = 3.6 times larger in intervention supermarkets (*B* = 1.28, *SE* = 0.512, *z* = 2.50, *p* = .012) than in control stores. However, their norm perceptions did not differ between intervention and control stores. In sum, a social normative intervention in a complex surrounding may impact purchasing behavior, but the mechanism driving this remains to be identified.

## INTRODUCTION

### Social norms and eating

Eating behavior is heavily subject to social influences (Herman et al., 2003). An important social influence affecting dietary decisions is the perception of social norms (Higgs, 2015). Social norms refer to an informal and implicit set of rules that describe what behavior is expected and appropriate in a particular context (Cialdini et al., 1990). According to the Focus Theory of Normative Conduct, social norm perceptions are commonly divided into two types based on different motivational pathways influencing behavior (Cialdini et al., 1990). Descriptive norms describe the behavior of others and serve as a heuristic, efficiently guiding individuals on how to behave, especially when uncertain of what behavior is considered appropriate in a specific context (Cialdini et al., 1990). Injunctive norms prescribe what others believe to be the socially correct or appropriate behavior in a given situation, and they are particularly followed to gain social approval (or avoid social disapproval) and to build and maintain relationships (Cialdini et al., 1990; Jacobson et al., 2011). Social norms are a powerful influence on eating behavior because of their adaptive function (Boyd et al., 2011; Higgs, 2015). That is, social norms can encourage beneficial eating behaviors, such as eating safe foods or adhering to table manners, preventing the need to individually find out what food is, for example, poisonous (Higgs, 2015). However, they can also perpetuate eating habits that harm human and planetary health.

One behavior that is strongly influenced by social norms and which is harmful to human and planetary health is meat consumption (Stoll‐Kleemann & Schmidt, 2017). Meat consumption, especially red and processed meats, is associated with increased risks of chronic diseases, like cardiovascular diseases, type 2 diabetes, and various types of cancer (Nelson et al., 2016). Additionally, meat production is a considerable driver of environmental breakdown, contributing to biodiversity loss, deteriorating water quality, and rising global greenhouse gas emissions (Willett et al., 2019). Therefore, to enhance both human and planetary health, it is crucial to shift diets from being predominantly animal‐based to predominantly plant‐based (Bui et al., 2024).

### Changing social norms

An important step in changing behavior maintained by social norms is to change the perceptions of social norms in favor of that behavior (Chen et al., 2024). Following the Focus Theory of Normative Conduct (Cialdini et al., 1990), social norms exert an influence on behavior only when this norm is salient (i.e., focal in attention) to an individual at the point of behavior. As such, to change norm perceptions and, consequently, behavior, a different or new social norm should become salient to the individual. That is, by highlighting a behavior performed by others (e.g., most other people are decreasing their meat intake), it is expected that the recipient of such a new norm may perceive meat reduction indeed as the common and appropriate behavior. Social norms can be made salient through textual communications (e.g., Robinson et al., 2014) or through the physical environment (e.g., Cialdini et al., 1990; Pechey et al., 2020; Raghoebar et al., 2020).

#### Textual norm communications

Textual communications can describe, for example, the behavior of the majority of a particular social group to communicate a descriptive norm (e.g., “80% of our customers purchase meat substitutes”). Recently, the textual communication of a dynamic descriptive norm has gained attention in the literature as it describes a trend or a change in behavior over time of a particular group (i.e., “More and more people are increasingly starting to limit their meat intake”; Sparkman & Walton, 2017; Sparkman et al., 2020). Dynamic norms are especially popular for stimulating pro‐environmental behaviors such as meat reduction or plant‐based product consumption, as they can highlight a majority without needing an actual majority to perform that behavior (Sparkman & Walton, 2017).

Several studies that textually communicated a dynamic norm have shown to be successful in reducing meat consumption or stimulating plant‐based food choices (intentions; De Groot et al., 2021; De Groot, 2022; Sparkman & Walton, 2017). However, the success of dynamic norm communications to improve sustainable consumption remains inconsistent, as some studies found no effects or counter‐effects (e.g., Aldoh et al., 2021, 2024). For example, in one of the studies of Sparkman et al. (2020), a dynamic norm communicated on a menu in a fine‐dining restaurant resulted in increased ordered meat dishes. Lastly, Coker et al., (2022) found no effect of dynamic norm communications on posters and banners in an in‐store restaurant.

#### Physical social norm communications

People also derive social norms from physical cues in the environment, such as open or closed lids and food traces that communicate that it is (un)acceptable to eat or choose a certain food (Raghoebar et al., 2019). Such environmental social norm cues have been shown to be predictive of people's eating behavior (Prinsen et al., 2013; Raghoebar et al., 2021). Social norms can also be communicated through the presence of certain foods or products in a given context or environment (Pechey et al., 2020). For example, an increased (absolute or relative) availability of a product in a supermarket may be interpreted by customers as a popular product high in demand, reflecting a descriptive norm perception (Pechey et al., 2019, 2020; van Kleef et al., 2012). A study by Raghoebar et al. (2020) showed that the increased availability of plant‐based foods (i.e., four plant‐based products to two meat products) in an imaginary supermarket setting influenced the descriptive norm perceptions, where people believed that the plant‐based option was commonly chosen.

Most studies investigating (dynamic) social norm interventions to stimulate meat reduction (intentions) or plant‐based consumption have been conducted either in (online) lab settings (e.g., Aldoh et al., 2024; De Groot et al., 2021; Sparkman & Walton, 2019) or relatively controlled environments, such as food environments with limited food options, like canteens and restaurants (e.g., Sparkman et al., 2020, 2021). It remains unclear to what extent such social norm interventions can stimulate meat substitute purchases in a more complex environment, such as real‐world supermarkets. Moreover, most of these studies measure (intended) behavior as an outcome yet do not take into account the extent to which these norm interventions result in stronger norm perceptions in intervention groups compared with control groups.

### Present study

The aim of the present study is to investigate if and how social norm communications favoring meat substitute purchases can shift norm perceptions and stimulate meat substitute purchases in a real‐world supermarket environment. Specifically, we aim to uncover the extent to which a social norm intervention in real‐world supermarkets stimulates meat substitute purchases and the extent to which perceived social norms favoring meat substitute purchases mediate this relationship.

The present study takes place in the Netherlands. Most people in the Netherlands eat meat (95%; CBS, 2023). While the Netherlands Nutrition Centre recommends a daily meat intake of 71 g (including 42 g of red meat), the average meat consumption in the Netherlands is 87 g of meat per day. People in the Netherlands eat meat on an average of five to six days per week (RIVM, 2024). In 2024, close to 70% of proteins purchased in supermarkets were animal‐based (Eiweet, 2024). Of the 32.4% products purchased that only contained plant‐based protein, only 5.4% came from products such as substitutes for meat, fish, dairy, and legumes. Meat, as well as meat substitutes, is mostly eaten at home (82%) and during dinner (71.9%; RIVM, 2024).

A 13‐week social norm intervention was implemented, including textual and physical norm communications, in three Dutch real‐world supermarkets, with three additional matched control supermarkets. Based on previous studies' findings that dynamic norm messages can strengthen norm perceptions (De Groot et al., 2021) and increased availability of plant‐based products can strengthen descriptive norm perceptions (Raghoebar et al., 2020), we hypothesize that:

Hypothesis 1 Perceptions of (a) descriptive, (b) injunctive, and (c) dynamic norms favoring meat substitute purchases will be stronger in the supermarkets with the social norm intervention compared with control supermarkets without the social norm intervention.

Furthermore, building on the predominantly positive findings of social norm interventions on meat reduction or substitution (De Groot, 2022; De Groot et al., 2021; Harguess et al., 2020; Kwasny et al., 2022; Sparkman & Walton, 2017), we hypothesize that:

Hypothesis 2 The supermarkets with social norm intervention, compared with the control supermarkets, will have (a) higher meat substitute purchases and (b) lower meat purchases.

Lastly, building on the notion that perceptions may mediate the relationship between social norm messages and behavior (Chen et al., 2024), we hypothesize that:

Hypothesis 3 The relationship between social norm intervention, compared with the control supermarkets, and meat substitute purchases is mediated by perceived (a) descriptive norms, (b) injunctive norms, and (c) dynamic norms.

## METHODS

### Study design and context

A quasi‐experimental design was used, including three intervention supermarkets and three matched control supermarkets, which are part of a supermarket chain in the Netherlands (with a market share of ~3.5%; ExpatINFO Holland, 2025; with a total of 121 shops in the Netherlands). A 13‐week intervention was implemented in the intervention supermarkets from September 5th, 2022, to December 4th, 2022. The three control stores continued business as usual during these weeks. The intervention stores were situated in the east, and the control stores were situated in the west of the Netherlands. The retailer selected control locations based on comparable sales trends for meat and meat substitutes, as well as their proximity to the nearest competitor, ensuring compatibility with the intervention locations. Communication about this intervention on (social) media (e.g., local newspaper) was intentionally restricted to prevent unintended influences on the results.

The intervention was an initiative of the General Affairs manager of the supermarket chain in partnership with a private organization that guides food services and retailers to improve the sustainability of their food supply. This private organization partnered with the supermarket chain to implement two interventions funded by the Dutch province of Gelderland: (1) reducing food waste and (2) promoting the protein transition by increasing plant‐based sales while decreasing meat sales. Their collaboration with the research team specifically focused on the second goal of the project. The academic research team of the present study supported the supermarket chain and private organization in the design of the (dynamic) social norm intervention and collected and analyzed the data for research purposes.

The effect of the social norm intervention on aggregated sales data at the supermarket level has been evaluated separately and presented elsewhere (Wolfswinkel, Raghoebar, et al., 2025). Ethical approval was granted by the Social Sciences Ethics Committee of Wageningen University, and the study was preregistered at Open Science Framework prior to data collection (OSF; https://osf.io/37rq8/?view_only=21e00a3ba70341acaaada47e2e613a05).

### Intervention elements and fidelity

**FIGURE 1:**
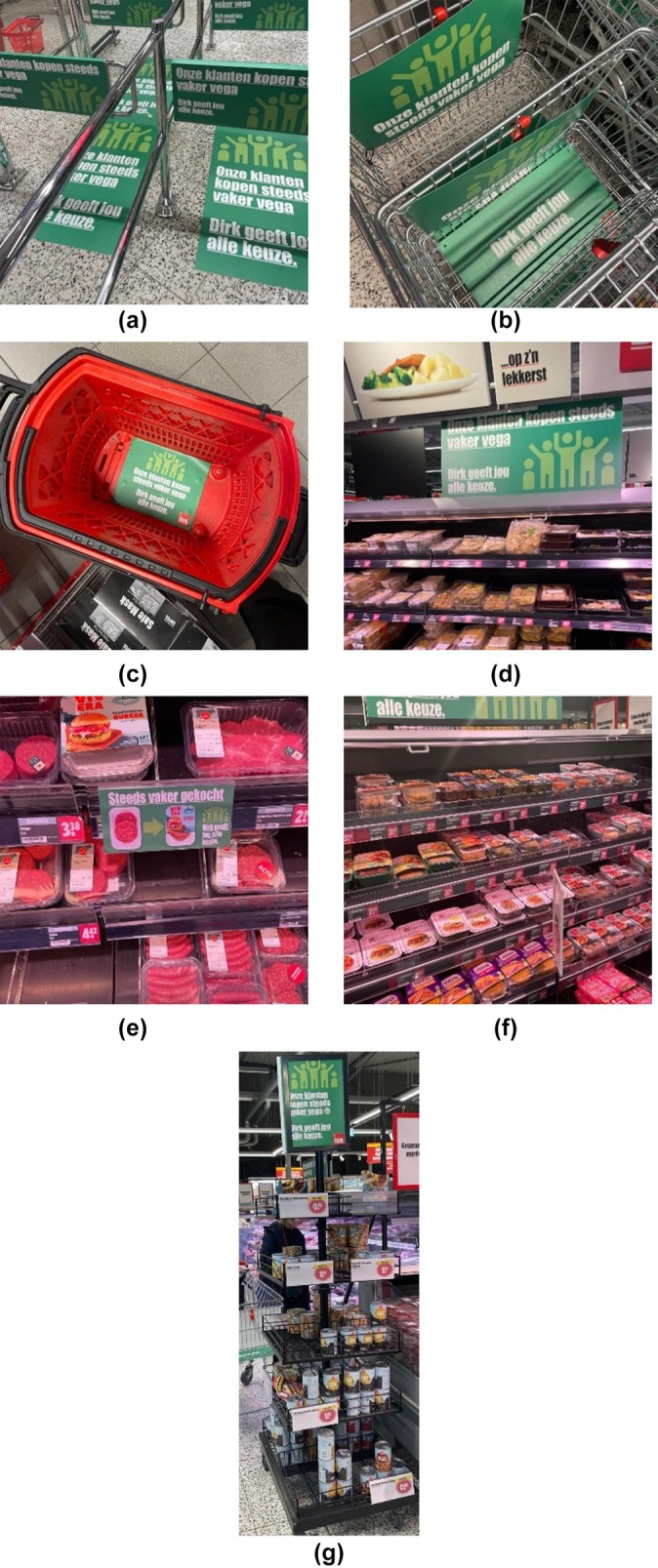
(a) Dynamic norm banner and floor sticker at supermarket entrance. (b) Dynamic norm banners in shopping carts. (c) Dynamic norm stickers in the shopping basket. (d) Dynamic norm banner above meat and meat substitutes in the meat aisle. (e) Dynamic norm wobbler between meat substitute and meat product. (f) Optically doubled meat substitutes to meat products. (g) Legume pop‐up island rack in the meat and meat substitute aisle.

The intervention consisted of textual dynamic norm communications and increased in‐store prominence of meat substitutes. The materials for the intervention were pretested among 30 customers in a supermarket location that was not part of the current study. The materials that were considered most appealing and yielded the least resistance were used for the eventual intervention. Banners, posters, and stickers were placed throughout the intervention supermarkets with a dynamic norm message: “Our customers are increasingly buying vegetarian – [supermarket name] offers a wide range of options.” This message was communicated on stickers on the floor at the entrance of the store, in the meat (substitute) aisle (Figure 1a), and in carts and baskets (Figure 1b,c). Banners were also placed in the meat aisle above the meat and meat substitutes (Figure 1d). Meat substitutes were placed next to the meat products, accompanied by a wobbler on the shelf with the message: “Increasingly purchased,” with a picture of a meat product on the left and a meat substitute on the right, and an arrow pointing from the meat to the meat substitute, suggesting that the meat substitute is being bought more often (Figure 1e).

To communicate the social norm promoting meat substitutes via the physical environment, the meat substitutes were positioned alongside meat in the same aisle and on the same shelf. Furthermore, the meat substitutes were displayed in a way that made their assortment appear visually larger than the meat selection (Figure 1f). Additionally, extra island racks featuring legumes (Figure 1g) were placed adjacent to the meat and meat substitute aisle, as well as in the regular canned food section of the supermarket, with the goal of increasing their prominence.

There were some discrepancies between the planned and actual implementation of the intervention. Banners and stickers were adjusted to include a plant‐based logo, differing from the original design; the correct versions were placed in the week of September 12th, 2022 (see Figure 1a–e). Due to logistical issues, dynamic norm messages were added to trolleys and baskets in the week of October 10th, 2022, without planned product images (https://osf.io/37rq8/?view_only=21e00a3ba70341acaaada47e2e613a05). In one supermarket, only a few carts featured these messages as the task was deemed too labor‐intensive by the store managers. Finally, shortages throughout the intervention period led to empty spots in the meat substitute section, with some plant‐based products occasionally replaced by meat to fill gaps (Wolfswinkel, Raghoebar, et al., 2025, for more details).

### Participants and study procedure

**FIGURE 2:**
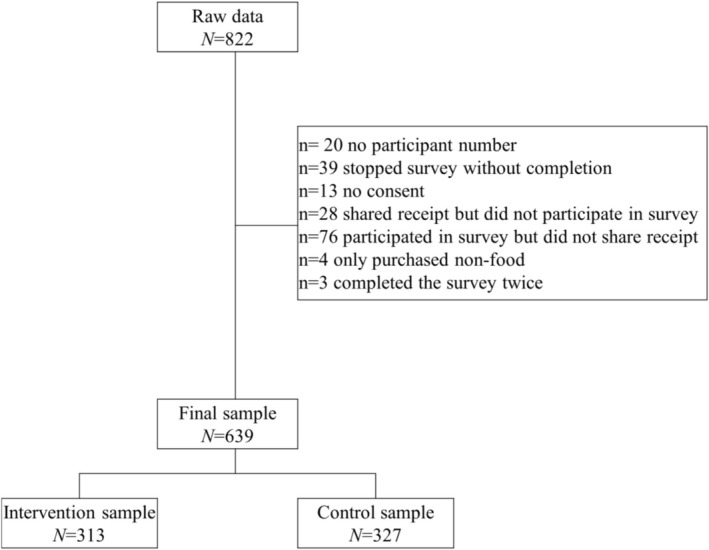
Participant flow chart.

Participants were recruited to fill out a survey and, if consent was provided, share their receipts. Power estimations were calculated before data collection and based on a Monte Carlo power analysis for indirect effects (mediation); a power of *β* = .80 was expected by an *N* = 570, *n* = 285 per condition (Raghoebar et al., 2020; Schoemann et al., 2017), also see preregistration (https://osf.io/37rq8/?view_only=21e00a3ba70341acaaada47e2e613a05). To account for dropouts or incomplete data, we aimed to recruit *n* = 110 per location. We ended up with a total of *N* = 822 participants who were recruited. Participants who only bought non‐food items were excluded from the data. After data cleaning, *N* = 639 participants filled out the survey and shared their receipts (Figure 2). The control condition consisted of *n* = 327 (51.1%) participants, and the intervention condition consisted of *n* = 313 (48.9%) participants.

During the second‐to‐last week of the intervention period (week of November 14th, 2022), adult participants who were proficient in Dutch were recruited by two researchers from the research team and/or employees of the private organization involved in the project (see 2.1). Data collection took place from Monday to Friday of that week, from 10 a.m. until approximately 3 p.m. when the maximum number of respondents for that location was collected. In all supermarkets, customers were approached after the check‐out and asked if they would be willing to participate in the study by filling out the survey about their experience with the supermarket and sharing their receipts. As an incentive, participants could partake in a raffle to win a €100 grocery voucher. The survey consisted of two parts, of which the first part was used for the present study. The second part of the survey was used for data collection of the private organization involved in the project and included questions about attitudes and preferences regarding healthy, sustainable, and vegetarian foods. Data for the second part of the survey were excluded from the present paper, as it was outside the scope of this paper. Recruited participants were assigned a unique participant ID number. The unique ID was used to connect survey data to the participants' receipts. However, as the receipt data and, thus, the unique ID were only necessary for the data collection of the present study, but not for the private organization, participants could still partake without a unique ID. When participants shared their receipts, a picture was taken of their receipts, including their participant ID number. Participants were then directed to a standing table specifically located near the exit of the supermarket for the purpose of this study, where they could fill out the online survey using one of the four research tablets or one of the two laptops. Alternatively, when all tablets/laptops were occupied or when the participant preferred, the survey could also be filled out on their own smartphone by scanning a QR code on posters that were located near the standing tables.

In the survey, participants were first asked to share their participant ID number. In case participants had a participant ID number, they would fill this out in the survey and continue to answer what store location they were in, and whether a photo was taken of their receipt. First, participants had to answer whether they had participated in this study before. If not, they could continue, and participants were asked for their informed consent, after which, if they agreed, the survey began.

In the first part of the survey, perceptions of social norms favoring meat substitute purchases, effort to purchase meat substitutes, and salience of meat substitutes were measured, followed by questions regarding participants' frequency of meat and meat substitute consumption in days per week, their dietary identity, for how many people and days they had bought groceries for, and questions to check if participants noticed a change in the supermarket. Lastly, they were asked to answer the descriptive variable questions (date of birth, gender, highest level of completed education, zip code). Once participants had finished, they were asked if they wanted to partake in the €100 grocery voucher raffle. If they did, they were asked to fill out their email address (voluntary).

### Measures

#### Outcome variables

The main outcome variables were the average grams of meat substitutes and meat purchased. Because most meat in the Netherlands is consumed during dinner (71.9%; RIVM, 2024), the intervention was targeted at increasing meat substitute purchases that are commonly eaten for dinner in the Netherlands. Therefore, meat substitutes included all uncooked vegan and vegetarian meat substitutes for at‐home preparation (e.g., veggie burgers, tofu, mock chicken) but excluded meat substitutes that are pre‐prepared breakfast or lunch deli meat substitutes (e.g., sandwich fillings like mock ham). Similarly, meat products included all uncooked meats and poultry (e.g., chicken breast and sausages), but excluded lunch or deli meats (e.g., sandwich fillings like chicken slices, bacon). Meat and meat substitute purchases were derived from the receipts that included data on the type, quantity (items purchased), weight (grams per item), and price of all purchased products. If the weight of the product was not on the receipt, it was looked up in the sales dataset provided by the retailer or on the website of the supermarket chain. Using pivot tables, the average grams of meat and meat substitutes were separately calculated for each participant.

#### Proposed mediators

The proposed mediator variables and related items are outlined in Table 1.^1^ Perceptions of descriptive and injunctive social norms favoring meat substitute purchases were modified based on the items by Raghoebar et al. (2020), and dynamic social norm perceptions favoring meat substitute purchases were inspired by Sparkman and Walton (2017). All items were measured on a 7‐point Likert scale ranging from 1 (*Totally disagree*) to 7 (*Totally agree*).

**TABLE 1:** Mediator variable items.

Variable	Items
Descriptive social norm	**Item 1.** It is likely that other customers of this [name supermarket] would purchase veggie* products. **Item 2.** It is common that other customers of this [name supermarket] would purchase veggie products.
Injunctive social norm	**Item 1.** Other customers of this [name supermarket] think you should purchase veggie products. **Item 2.** Other customers of this [name supermarket] think it is appropriate that you purchase veggie products.
Dynamic social norm	**Item 1.** An increasing number of customers of [name supermarket] are starting to purchase veggie products. **Item 2.** Customers of [name supermarket] are increasingly purchasing veggie products.

*Note*: “Veggie” in this case translates to “vega” in Dutch, which is a common abbreviation for “vegetarian” or “meat substitute.” “Veggie” (“vega” in Dutch) was explained in the survey.

#### Descriptive variables

Participant's dietary pattern was measured with the question: “Which dietary pattern describes yours most accurately?” with five answer options (i.e., meat eater, flexitarian [sometimes meat], pescetarian [no meat, only fish], vegetarian [no meat or fish, only eggs and dairy such as cheese and milk], vegan [no meat, no fish, no eggs, and no dairy such as cheese and milk]). Participants' frequency of meat and meat substitute consumption was measured with two questions: “How many days a week do you typically eat meat/meat substitutes?” (never, 1 day, 2 days … 6 days, every day). Participants could indicate how many people they had bought groceries for (open‐ended question) and for how many days they had purchased groceries (1 to 7 days). Participant's date of birth (DD/MM/YYYY) was used to determine their age at the time of data collection. Gender was measured by asking how participants identified: with five options (i.e., woman, man, non‐binary, something else, prefer not to say). Level of education was measured with: “What is your highest level of completed education?” with seven options (i.e., no education, elementary school, pre‐vocational secondary education, higher general continued education or preparatory scientific education, secondary vocational education, higher professional education, and academic education). The seven options were categorized into three levels of education (“lower, middle, higher”) based on the categorization of Statistics Netherlands (CBS, 2018). The participant's zip code was asked to determine neighborhood‐level social security benefits (i.e., the number of people in that zip code area that receive social security benefits below the age of retirement), with higher numbers representing lower household income levels (Centraal Bureau voor de Statistiek, n.d.).

#### Intervention (element) awareness

To check the extent to which participants noticed the supermarket intervention, the following question was asked: “Is there something that you noticed while you were doing your groceries at [name supermarket]?” (yes/no). If participants indicated “yes,” they were asked to write down what they noticed to identify if the intervention (or elements) were recognized as such (yes/no).

### Data analyses

#### Sample characteristics and internal consistency

Descriptive statistics (M, SD, or *n*, %) were assessed for all variables, including gender, age, education level, dietary pattern, meat and meat substitute consumption in days a week, number of days groceries were bought for, and the intervention awareness. One‐way ANOVAs and Chi‐square tests were conducted to determine whether these variables differed between the intervention and control supermarkets, except for meat and meat substitute purchases. For meat and meat substitute purchases, the median and interquartile range were calculated among non‐zero data because they were zero‐inflated. A Mann–Whitney *U* test was used to assess the difference between the control and the intervention group.

All two‐item variables were tested for internal consistency using the Spearman–Brown coefficient (Eisinga et al., 2013). Values ≥.70 were considered sufficient to merge the items into one variable (de Vet et al., 2017). The Spearman–Brown coefficient was calculated for descriptive norm perceptions (Spearman–Brown = .770), injunctive norm perceptions (Spearman–Brown = .744), and dynamic norm perceptions (Spearman–Brown = .866).

#### Main analysis

Separate linear regressions were performed to test whether the mediators (Hypothesis 1)—perceived descriptive, injunctive, and dynamic social norms favoring meat reduction—differed between intervention and control stores. These linear regressions also functioned to check whether the conditions for mediation analysis (Hypothesis 2) were met. Additionally, to check whether the conditions for mediation analysis (Hypothesis 2) were met, separate linear regressions were performed to assess the relation between each proposed mediator and the primary outcome variable (i.e., meat substitute purchases). Proposed mediators that showed a significant association in both pathways were eligible to be included in the causal mediation analysis. Two separate regressions were conducted to test for a difference in meat substitute purchases (Hypothesis 3a) and meat purchases (Hypothesis 3b) between the intervention and control stores. To deal with the zero‐inflated outcome variable of meat and meat substitutes, a Two‐Part model was used with the *glmmTMB* package in *R*, in which the first part (*Part I*) of the model describes the probability of meat (substitute) purchases being nonzero and the second part (*Part II*) of the model describes the magnitude of the nonzero values (i.e., the amount of meat substitutes purchased in grams among the participant that purchased meat substitutes; Liu et al., 2019; van der Vliet et al., 2024).

#### Sensitivity and exploratory analyses

The same linear regression was used to test Hypothesis 3 to examine the extent to which the intervention effect on meat substitute purchases changed when controlling for age, gender, education, neighborhood social security benefits, and intervention awareness. Also, the intervention effect on meat substitutes was assessed, excluding all vegetarian, pescatarian, and vegan participants, as it was expected that those might have been less affected by the intervention. Exploratory moderation analyses were conducted to investigate the extent to which the relationship between perceptions of descriptive, injunctive, and dynamic norms and the (likelihood of) meat substitute purchases differed between the intervention and the control stores.

## RESULTS

### Sample characteristics

The majority of the participants were female (73.7%, *n* = 471), the average age was 60.8 (SD = 14.6) years, and 40.7% (*n* = 260) of the sample had completed a lower level education, followed by middle (*n* = 235, 36.8%), and higher level education (*n* = 144, 22.5%).^2^ Most of the participants (*n* = 351, 54.9%) described themselves as meat eater, followed by 39.1% (*n* = 250) as flexitarians, 3.1% (n = 20) as vegetarians, 2.0% (*n* = 13) as pescatarians, and 0.8% (*n* = 5) as vegans. Participants indicated that they ate meat on 3.9 (SD = 1.8) days a week and were vegetarian on 1.4 (SD = 1.6) days a week. There were no significant differences between the control and intervention conditions in age, gender, education, dietary identity, meat and meat substitute consumption in days a week, and the number of people the groceries were bought for (Table 2). The number of days the groceries were bought for was significantly higher in the intervention group (Table 2). There was no significant difference in whether participants noticed something in the supermarket between the two conditions. The majority of participants, in the control (77.3%, *n* = 242) and in the intervention (73.9%, *n* = 241) group, indicated not having noticed a difference in the store (*χ* ^2^(1, 639) = .319).

**TABLE 2:** Descriptive statistics for total sample, control, and intervention group.

	Total (*N* = 639)	Control (*n* = 313)	Intervention (*n* = 326)	Test	Effect size	*p*
Age (mean, SD)	60.8(14.6)	60.4(14.9)	61.1(14.4)	*F*(1, 632) = 0.337	*η* ^2^ = .001	.562
Gender (female) (*n*, %)	73.7% (471)	72.8% (228)	74.5% (243)	*χ* ^2^(3, 639) = 2.04	Cramer's *V* = .056	.565
Education, *n* (%)				*χ* ^2^(3, 639) = 3.89	Cramer's *V* = .078	.143
Lower level education	40.7% (260)	44.4% (139)	37.1% (121)			
Middle‐level education	36.7% (235)	33.5% (105)	39.8% (130)			
Higher level education	22.5% (144)	22.0% (69)	23.0% (75)			
Dietary identity				*χ* ^2^(4, 639) = 2.36	Cramer's *V* = .061	.670
Omnivore	54.9% (351)	54.0% (169)	55.8% (182)			
Flexitarian	39.1% (250)	39.3% (123)	39.0% (127)			
Vegetarian	3.1% (20)	3.5% (11)	2.8% (9)			
Pescetarian	2.0% (13)	1.9% (6)	2.1% (7)			
Vegan	0.8% (5)	1.3% (4)	0.3% (1)			
Meat consumption frequency^a^	3.9 (1.8)	3.9 (1.8)	4.0 (1.8)	*F*(1, 637) = 0.793	*η* ^2^ = .001	.374
Meat substitute consumption frequency^a^	1.4 (1.6)	1.3 (1.5)	1.4 (1.7)	*F*(1, 637) = 0.831	*η* ^2^ = .001	.569
Descriptive norm perceptions	4.2 (1.5)	4.3 (1.5)	4.1 (1.5)	*F*(1, 637) = 2.8	*η* ^2^ = .004	.093
Injunctive norm perceptions	3.6 (1.6)	3.7 (1.6)	3.6 (1.6)	*F*(1, 637) = 1.7	*η* ^2^ = .003	.197
Dynamic norm perceptions	4.1 (1.6)	4.2 (1.6)	4.1 (1.6)	*F*(1, 637) = 0.3	*η* ^2^ = .000	.601
Meat substitute purchased in grams				*z* = −2.674	*η* ^2^ = .011	.007
Zero‐inflation percentage	96.4%	98.4%	94.5%			
Median purchases (IQR)^b^	180.0 (245.0)	180.0 (200.0)	240.0 (383.8)			
Meat purchased in grams				*z* = −0.662	*η* ^2^ = .000	.508
Zero‐inflation percentage	62.1%	60.7%	63.5%			
Median purchases (IQR)^b^	550.0 (578.3)	574.0 (668.0)	530.0 (568.0)			
Number of people's groceries done for	2.5 (1.7)	2.4 (1.3)	2.6 (2.0)	*F*(1, 637) = 0.881	*η* ^2^ = .001	.348
Number of days groceries are done for	2.8 (1.9)	2.6 (1.8)	3.0 (1.9)	*F*(1, 637) = 7.298	*η* ^2^ = .011	.007

^a^ In days a week.

^b^ Among nonzero data.

From the participants who did notice a change in the supermarket, a total of 87 observations were described across *n* = 70 participants in the intervention group, and 68 observations were described across *n* = 64 participants in the control group. Of the 70 participants (16.1%) who noticed a change in the supermarket in the intervention store, 13.8% (*n* = 12) mentioned intervention elements, which is 3.7% of all participants in the intervention stores. None of these participants bought meat substitutes. Other mentioned aspects included: a busy or crowded store (*n* = 16, 18.4%) and missing products or empty shelves (13.8%, *n* = 12). In the control group, the presentation or salience of meat substitutes was mentioned by 4.4% (*n* = 3). The two most mentioned observations in the control group were missing products or empty shelves (*n* = 17, 25%) and general satisfaction with products, the store, and/or staff (*n* = 10, 14.7%).

### Main analysis

#### Intervention effect on mediators

There were no differences in descriptive (*β* = −.07, *SE* = 0.12, *t* = −1.68, *p* = .094, 95% CI[−0.30, 0.17]), injunctive (*β* = −.05, *SE* = 0.13, *t* = −1.29, *p* = .197, 95 CI[−0.30, 0.20]), and dynamic (*β* = −.02, *SE* = 0.13, *t* = −0.523, *p* = .601, 95% CI[−0.27, 0.23]) norm perceptions favoring meat substitute purchases between the intervention and control stores.

#### Mediator effects on main outcomes

Descriptive, injunctive, and dynamic social norm perceptions favoring meat substitute purchases did not affect the likelihood or amount of meat substitute purchases (Table 3). Therefore, together with the null effect of the intervention on the norm perceptions (Section 3.2.1), the conditions to conduct mediation analysis were not met.^3^

**TABLE 3:** Two‐part models for the effect of mediators on meat substitute purchases.

	*B*	*SE*	*z*	*p*	95% CI	
Descriptive norm perception						
Part I	0.034	0.142	0.236	0.814	−0.24	0.31
Part II	0.152	0.119	1.281	0.200	−0.08	0.38
Injunctive norm perception						
Part I	−0.002	0.133	−0.015	0.988	−0.26	0.26
Part II	0.091	0.127	0.712	0.477	−0.16	0.34
Dynamic norm perception						
Part I	0.150	0.138	1.088	0.277	−0.12	0.42
Part II	−0.014	0.085	−0.165	0.869	−0.18	0.15

#### Intervention effect on meat substitute and meat purchases

**FIGURE 3:**
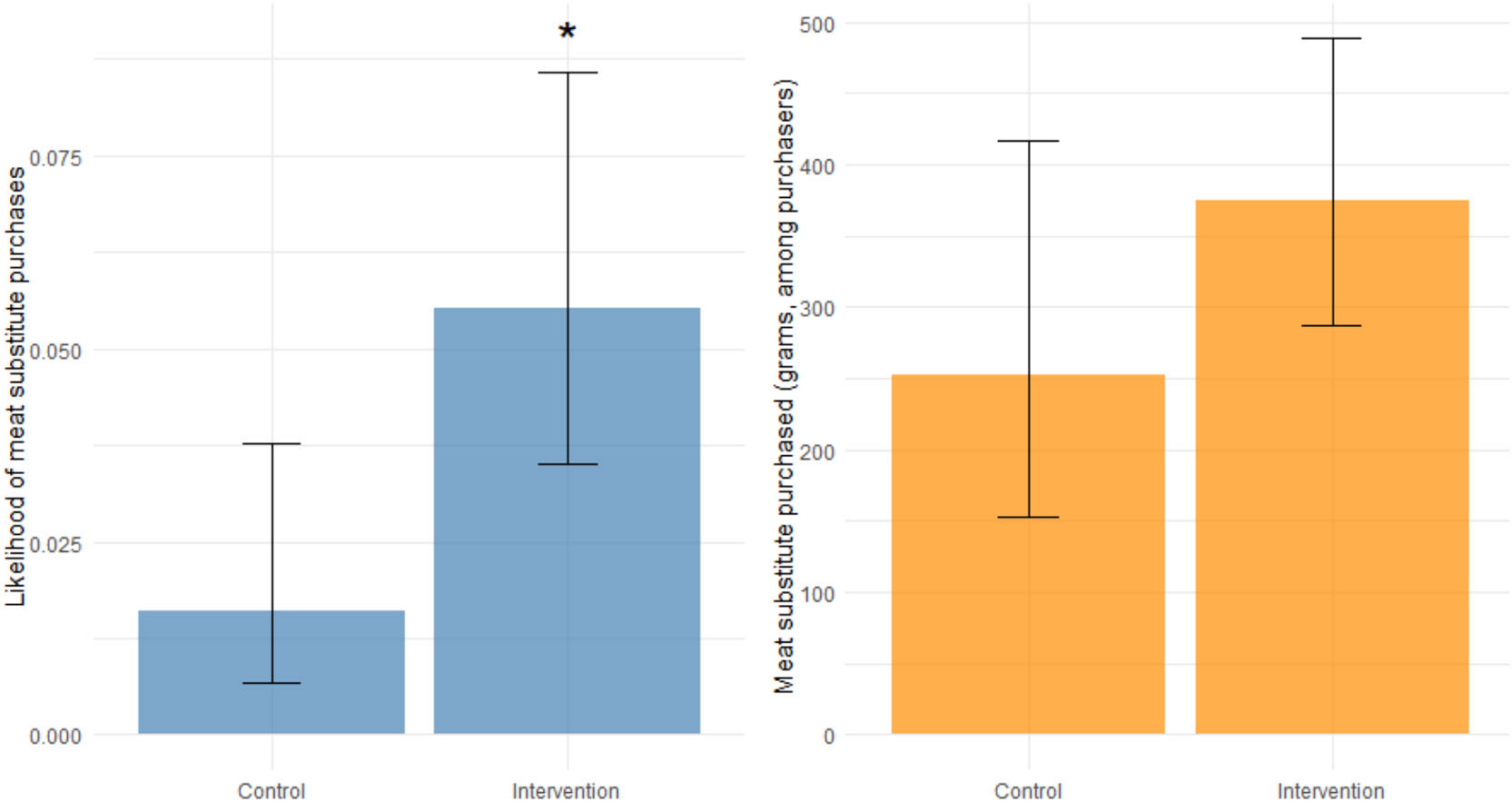
Visual presentation of the difference in likelihood of meat substitute purchases (left) and amount of meat substitute purchases between intervention and control stores.

The median grams of meat substitutes purchased (excluding zero‐inflation) in the intervention stores was *Median* = 240.0 (*IQR* = 383.8; *n* = 18; 5.5%), and *Median* = 180.0 (*IQR* = 200.0; *n* = 5; 1.6%) in the control stores. The first part of the model (*Part I*) shows that the intervention had a significant effect on the likelihood of meat substitutes being purchased (*B* = 1.28, *SE* = 0.512, *z* = 2.50, *p* = .012, 95% CI[0.28, 2.28]), exponentiation of the coefficient indicates that participants in the intervention stores were 3.6 times more likely to purchase a meat substitute compared with participants in the control stores (Figure 3). However, while descriptively more meat substitutes (in grams) were purchased in the intervention condition, the second part of the model (*Part* II) shows that among those participants that did purchase meat substitutes, the amount purchased (grams) did not differ significantly (*B* = 0.40, *SE* = 0.290, *z* = 1.37, *p* = .172, 95% CI[−0.17, 0.97]; Figure 3). The median grams of meat purchased in the intervention stores was *Median* = 530.0 (*IQR* = 568.0; *n* = 123; 39.3%), and *Median* = 574.0 (*IQR* = 668.0; *n* = 119; 38.0%) in the control stores. The intervention did not affect the likelihood of meat purchases (*B* = −0.12, *SE* = 0.16, *z* = −0.728, *p* = .467, 95% CI[−0.44, 0.20]), nor the amount of weight of meat purchases among those that purchased meat (*B* = 0.00, *SE* = 0.08, *z* = 0.04, *p* = .970, 95% CI[−0.16, 0.16]).

The effect of the intervention on the likelihood of meat substitute purchases remains significant when controlling for the number of days and people groceries were bought for, age, gender, education, and neighborhood social security benefits (Table 4). Lastly, the intervention effect of the likelihood of meat substitutes purchased remained significant when all vegetarian, pescatarian, and vegan participants (*n* = 38) were excluded from the analyses (*B* = 1.59, *SE* = 0.638, *z* = 2.50, *p* = 0.013).

**TABLE 4:** Two‐part model for sensitivity analysis of condition effect on meat substitute purchases.

	*B*	*SE*	*z*	*p*	95% CI	
*Part I*						
Condition (intervention)	1.085	0.530	2.048	.041	0.05	2.12
Number of days groceries are done for	0.197	0.107	1.844	.065	−0.01	0.41
Number of people's groceries done for	−0.153	0.184	−0.829	.407	−0.51	0.21
Age	−0.016	0.016	−1.023	.306	−0.05	0.01
Gender (male)	−0.807	0.646	−1.249	.212	−2.07	0.46
Education (middle level)	0.669	0.578	1.158	.247	−0.46	1.80
Education (high level)	0.831	0.652	1.276	.202	−0.45	2.11
Social security benefits the neighborhood	0.000	0.001	0.503	.615	0.00	0.00
*Part II*						
Condition (intervention)	0.030	0.281	0.107	.915	0.52	0.58
Number of days groceries are done for	0.077	0.045	1.703	.089	−0.01	0.17
Number of people's groceries done for	0.054	0.138	0.39	.696	−0.22	0.32
Age	−0.010	0.008	−1.360	.174	−0.03	0.00
Gender (male)	0.895	0.488	1.836	.066	−0.06	1.85
Education (middle level)	0.101	0.288	0.350	.727	−0.46	0.67
Education (high level)	0.528	0.304	1.738	.082	−0.07	1.12
Social security benefits the neighborhood	0.000	0.001	−0.478	.632	0.00	0.00

*Note*: As the number of days that people bought groceries was the only covariate that was significantly different between the conditions, we also tested this variable as a singular covariate. The results remained the same, controlling for only the number of days groceries were done: Part I (*B* = 1.19, *SE* = 0.52, *z* = 2.31, *p* = .021, 95% CI[0.18, 2.20]), Part II (*B* = 0.21, *SE* = 0.30, *z* = 0.687, *p* = .492, 95% CI[−0.39, 0.19]).

## DISCUSSION

The aim of the present study was to investigate whether social norm communications (i.e., textual and physical) affect meat and meat substitute purchases in intervention stores compared with control stores, and to what extent this relation is mediated by perceived social (i.e., descriptive, injunctive, and dynamic) norms favoring meat substitute purchases. Contrary to our expectations, we found no support for the hypothesis that perceptions of social norms encouraging meat substitutes were stronger in supermarkets with a social norm intervention favoring meat substitutes compared with control supermarkets (Hypothesis 1). Neither did we find support that perceptions of social norms were associated with meat substitute purchases. Nevertheless, we did find support for the hypothesis that the social norm intervention would increase the likelihood of meat substitutes being purchased in the intervention supermarkets (Hypothesis 2). This partial intervention effect could, however, not be explained by stronger social norm perceptions in the intervention stores compared with the control stores, nor by stronger perceptions of effort and salience favoring meat substitutes (Hypothesis 3).

The lack of difference in norm perceptions between intervention and control stores, as well as the lack of association between norm perceptions and (likelihood of) meat substitute purchases, contradict earlier findings in more controlled environments, such as (online) lab settings (De Groot, 2022; De Groot et al., 2021; Sparkman & Walton, 2017). Specifically, these studies showed increased norm perceptions favoring meat reduction (e.g., De Groot et al., 2021) and increased (intention to or interest in) plant‐based food choices or meat reduction (De Groot, 2022; De Groot et al., 2021; Edwards et al., 2026; Sparkman & Walton, 2017).

An explanation for the absence of differences in norm perceptions between the intervention and control supermarkets may be that the social norm intervention was not salient (enough) for customers for their norm perceptions to be affected. This is also reflected in the lack of differences between intervention and control stores in the relationship between social norm perceptions and (likelihood of) meat substitute purchases. Following the Focus Theory of Normative Conduct (Cialdini et al., 1990), a norm should be salient (i.e., focal in attention) to affect behavior. A noteworthy strength of the present study is that we conducted the intervention in real‐world supermarkets, including actual behavior (opposed to self‐reported or intended behavior). A downside, however, is the noisiness of the environment of supermarkets (Thompson et al., 2013). Supermarkets are known for being a complex food environment due to the variety of external cues demanding consumers' attention (i.e., music, [price] promotions, scent, lighting, etc.; Thompson et al., 2013). The overwhelming external cues dragging the consumer's attention may explain why the perceptions were not different between conditions. This is also underscored by the results of the manipulation check, where only a small minority noticed the intervention elements in the intervention stores. Consequently, the social norm intervention may not have been (deliberately) processed to customers. Unexpectedly, given the lack of differences in norm perceptions between intervention and control stores, the receipts indicated a higher likelihood of meat substitute purchases in the intervention supermarkets compared with the control supermarkets, partially supporting our second hypothesis. Earlier studies implementing social norm interventions to stimulate plant‐based food choices have resulted in null results (Aldoh et al., 2021, 2024), especially in more complex food environments such as (in‐store) restaurants (Coker et al., 2022; Sparkman et al., 2020). Moreover, a recent study also showed increased norm perceptions but unaffected intended behavior after a dynamic social norm message (Wolfswinkel, Sparkman, et al., 2025). This raises the question: why was the likelihood of meat substitutes being purchased higher in intervention supermarkets compared with control supermarkets, but not social norm perceptions?

One explanation may relate to people's norm perceptions. Descriptively but not significantly, these were equal or stronger in control stores than in intervention stores. This could imply that any intervention effects may have been offset by stronger perceptions in the control stores. This difference in potential perceptions may have been caused by the regional differences between the intervention and control store locations. There is a substantial difference in meat consumption between Dutch regions, ranging from 37% to 65% of people within that region eating meat on more than five days a week (CBS, 2023). The intervention stores were located in the east of the Netherlands (Province of Gelderland) and the control stores in the west of the Netherlands (provinces of North and South Holland), situated close to two of the largest cities of the Netherlands (i.e., Amsterdam and Rotterdam). People in the region where the intervention stores were located eat meat more frequently compared with the region where the control stores were located (CBS, 2023). These regional differences may have possibly led to stronger baseline perceptions in control stores and should be considered when interpreting results. However, as the current study design did not include pre‐ and post‐tests of norm perceptions in the intervention and control stores, we lack insight into whether and how norm perceptions changed during the intervention period.

Moreover, the intervention may have triggered mechanisms responsible for the intervention effect that were unrelated to social norms, effort, and salience. It could, for instance, be that the intervention influenced perceptions of tastiness, self‐efficacy for preparing plant‐based meals, or that it triggered novelty‐seeking motives. This may also provide an explanation for the lack of association between norm perceptions and likelihood of meat substitute purchases.

Notably, the intervention effect on individual purchase behavior observed in the present study contradicts the findings from the evaluation of its effect on aggregated sales data. The social norm intervention of the present study was also evaluated on its effect on aggregated sales data at the supermarket level and presented in Wolfswinkel, Raghoebar, et al. (2025). Similar to the present study, the outcomes were the average sales of meat substitutes, legumes, and meat in grams per week, but showed that the meat substitute purchases in grams per week were unaffected in the intervention stores compared with the control stores and the pre‐intervention sales trends (Wolfswinkel, Raghoebar, et al., 2025). Taken together with the results of the present study, it seems that only a subgroup of people were affected by the intervention in terms of their purchasing behavior. This discrepancy may be explained by pre‐existing preferences toward meat substitutes that may have enhanced an intervention effect. Namely, (social norm) nudges have been reported to be especially effective when people do not have a strong preference for or against the targeted behavior (e.g., meat substitute purchases; De Ridder et al., 2022; Venema et al., 2019, 2020). There is a general tendency that consumers are skeptical of meat alternatives in Western countries (Wassmann et al., 2025), as they are considered less healthy, relatively expensive, and at times even less environmentally friendly compared with meat (Wassmann et al., 2025). This skepticism was also apparent from the participant recruitment, as some participants walked away annoyed because the survey questions mentioned meat substitutes (although we do not have exact records of this). It is possible that, because of the voluntary nature of participating in the survey, people in both the control and intervention groups may have initially been neutral or slightly open to meat substitutes compared with the general sample, as customers with negative attitudes or preferences toward meat substitutes may have felt less inclined to participate.

### Strengths and limitations

A strength of the present study is that we conducted an experiment in real‐world supermarkets, measuring actual behavior (i.e., meat substitute purchases), while also investigating to what extent it would lead to stronger norm perceptions in the intervention condition compared with the control condition. To the best of our knowledge, this is the first study to measure both an intervention effect targeting actual meat (substitute) purchases and the associated norm perceptions in a complex real‐world context. Moreover, it was intentionally decided to omit (social) media communications regarding the intervention until after the intervention to prevent influencing customers during the intervention period.

Some limitations need to be mentioned. First, we lack insight into how representative our sample of supermarket customers is in this study, as we do not have information about the customer profiles of the supermarkets. Moreover, the implementation fidelity of the intervention left something to be desired (van Rookhuijzen & de Vet, 2021; Vogel et al., 2023). However, relatively low implementation fidelity is typical in complex, real‐world environments like supermarkets (van Rookhuijzen & de Vet, 2021; Vogel et al., 2023). Nevertheless, a more systematic or quantitative approach to fidelity assessment could have strengthened the reporting in this study. Furthermore, all data collection happened between 10 a.m. and approximately 3 p.m. on weekdays at all locations. However, the days of data collection were not paired between control and intervention stores (e.g., data collection of intervention store on Monday paired to data collection of control store on Monday). Moreover, the participants of the present study are a specific group of people who shop during these hours, including predominantly women who are 60 years old on average. The overrepresentation of women in this sample can be considered representative, as women are responsible for grocery shopping in the Netherlands (Roeters, 2019). But the relatively higher age of the sample could be attributed to the data collection period within work hours. Nevertheless, we lack insight into the demographics of intervention and control stores. Therefore, it remains unclear to what extent the sample is representative in terms of the customers of the participating stores. This may have affected the results, as this could have been a group less open to meat substitutes. Specifically, younger populations eat substantially more meat substitutes compared with people older than 51 years in the Netherlands (RIVM, 2024). Lastly, implementing the intervention in a different region of the Netherlands than the control supermarkets reduced the risk of people in the control condition accidentally being exposed to the intervention. However, as meat consumption differs regionally in the Netherlands, this may have affected the results.

### Recommendations for future research

The present study suggests that although a social norm intervention may increase the likelihood of customers purchasing meat substitutes, the intervention did not lead to stronger norm perceptions favoring meat substitute purchases in the intervention stores. The results of this study raise multiple questions. First, while the intervention effect was only partial, it could not be explained by social norm perceptions or perceptions of effort and salience. This raises the question what mechanism could be driving this effect? Future research could also take into account other potentially explanatory measures such as self‐efficacy or expected tastiness of meat substitutes (Stoll‐Kleemann & Schmidt, 2017). Moreover, a recent study found that dynamic norm messages may temper the effect of strong meat‐eating habits and that they may specifically increase intentions to reduce meat consumption among individuals with weaker attachment to meat (Wolfswinkel, Sparkman, et al., 2025). Therefore, it may be worthwhile to include measurements of meat‐eating habit strength and meat attachment in future studies. Second, to what extent was the intervention both salient and long enough to shift perceptions of social norms? To date, little is known about how long it takes until norm perceptions shift. Future research could investigate social norm shifts longitudinally to shed light on potential social norm perception tipping points. Related to this, we did not measure baseline perceptions of social norms, effort, and salience, as we did not want to influence customers prior to or during the intervention. It would, however, be interesting to know if perceptions within the intervention stores changed at all. Recent findings of a correlational study showed that social norm perceptions favoring both meat consumption and meat reduction differ across food environments (Wolfswinkel et al., 2024). It would be an interesting angle for future research to include a baseline measurement of norm perceptions to measure if and how they change over time. Further, future supermarket interventions may benefit from a more structured and extensive approach to fidelity assessment. This could aid in quickly correcting mistakes or miscommunications between the involved partners (e.g., research team, retailer's head office, supermarket employees). Additionally, the present study found a modest partial intervention effect of increased likelihood of meat substitute purchases in the intervention stores. This is promising in showing that subtle interventions communicating a social norm textually and physically may stimulate plant‐based choices in complex environments. However, the lack of difference in norm perception between conditions, together with the aggregated sales data (Wolfswinkel, Raghoebar, et al., 2025), suggests future supermarket interventions may benefit from (additional) structural measures (Adams et al., 2016). For example, combining a social norm intervention with carbon taxes on meat products, as the latter has been shown to be promising in reducing greenhouse gas emissions in simulation studies (Pinto, 2021). Moreover, while we did not record this, incidentally, participants verbally expressed they felt annoyed by the topic of meat substitutes. Incorporating qualitative methods to assess customer experiences and perceptions regarding (social norm) interventions aimed at increasing meat substitute purchases could further enrich our understanding of customers' acceptance of such interventions. Lastly, as the intervention consisted of multiple elements, we deliberately asked participants a broad manipulation check question (i.e., “Did you notice a change in the supermarket?” and let participants share what they noticed). Due to the busy nature of supermarkets, it is, however, possible that participants did not recall the intervention elements at the point of measurement. Future research could incorporate more specific manipulation check questions related to different intervention elements to further narrow down if and what participants noticed.

### Concluding remarks

The present study offers a novel contribution to social norm literature by conducting a social norm intervention in a complex real‐world supermarket, measuring actual behavior and perceptions of social norms, effort, and salience related to meat substitute purchases. The results showing an increased likelihood of meat substitute purchases in the intervention stores seem promising. We were, however, unable to explain this increased likelihood through strengthened social norm perceptions (or perceptions of effort and salience). Moreover, the findings of the present study contradict findings on the intervention effect on aggregated sales data. Taken together, these findings raise questions about the duration and salience of social norm interventions needed to shift perceptions and fruitful methods to capture potential perception shifts in complex food environments such as supermarkets. Yet, the findings of the present study also open doors to the potentially successful effect of social norm interventions in supermarkets aimed at stimulating meat substitute purchases.

## AUTHOR CONTRIBUTIONS

All authors contributed to the design of the study, and all authors contributed to the writing and reviewing of the manuscript. S.W. conducted the data analysis. All authors read and approved the final manuscript.

## CONFLICT OF INTEREST STATEMENT

Nothing to disclose.

## ETHICS STATEMENT

Ethical approval was granted by the Social Sciences Ethics Committee of Wageningen University (reference code: 2022–93‐Wolfswinkel).

## Supporting information


**Table S1.** Variable Items for Perceived Effort and Perceived Salience of (Purchasing) Meat Substitutes.
**Table S2.** Two‐Part Models for Effect of Perceived Effort and Perceived Salience on Meat Substitute Purchases.

## Data Availability

The data that support the findings of this study are available from the corresponding author upon reasonable request.

## Footnotes

^1^
To account for a potential alternative explanation of the expected intervention effect (i.e., increased meat substitute purchases through increased norm perceptions), we also tested perceived salience of meat substitutes and perceived effort to purchase as potential mediators of the intervention effect on meat substitute purchases. We found no effects of the intervention on perceptions of effort and salience, neither did perceptions of effort and salience affect meat substitute purchases. See Appendix S1 for a short report.

^2^
A lower level education refers to no education, primary school, or prevocational secondary education. Middle level education refers to senior general secondary education and preuniversity education. A higher level education refers to tertiary education.

^3^
Exploratory analysis revealed that there was no difference between the intervention and control stores in the relationship between the likelihood of meat substitute purchases and the perception of descriptive (*B* = 0.18, *SE* = 0.338, *z* = 0.520, *p* = .603, 95% CI[−0.49, 0.84]), injunctive (*B* = −0.12, *SE* = 0.321, *z* = −0.383, *p* = .702, 95% CI[−0.75, 0.51]), or dynamic (*B* = 0.30, *SE* = 0.323, *z* = 0.940, *p* = .347, 95% CI[−0.33, 0.94]) social norms. There was also no difference between the intervention and control stores in the relationship between the amount of meat substitute purchases in grams and the perception of descriptive (*B* = −0.17, *SE* = 0.283, *z* = −0.585, *p* = .559, 95% CI[−0.72, 0.39]), injunctive (*B* = 0.83, *SE* = 0.779, *z* = 1.066, *p* = .286, 95% CI[−0.70, 2.36]), or dynamic (*B* = 0.02, *SE* = 0.235, *z* = 0.065, *p* = .948, 95% CI[−0.44, 0.47]) social norms.
