# Supplementary material for: Social norm intervention in real‐world supermarkets in the Netherlands: A quasi‐experimental evaluation of effects on perceived social norms and meat substitute purchases
Source: Appl Psychol Health Well Being. 2026 May 8;18:e70159. doi: 10.1111/aphw.70159 (PMC13155190; doi:10.1111/aphw.70159)
Supplement: Supplementary file 1 — Table S1. Variable Items for Perceived Effort and Perceived Salience of (Purchasing) Meat Substitutes. Table S2. Two‐Part Models for Effect of Perceived Effort and Perceived Salience on Meat Substitute Purchases. [file APHW-18-0-s001.docx]

**Appendix A**

A1. Background and Hypotheses

In the present study, we hypothesize that the social norm intervention implemented in the present study would affect meat substitute purchases through the perception of social norms. However, such an intervention may also affect meat substitute purchases because the intervention increases the salience of meat substitutes, and decreases the effort it takes to purchase meat substitutes. To account for this alternative explanation, we included perceived effort of purchasing meat substitutes and perceived salience of meat substitutes as alternative or additional mediators. It was hypothesized that:

HA1 The presence of the social norm intervention, compared to the absence in the control supermarkets, will lead to stronger perceived salience of meat substitutes;

HA2 The presence of the social norm intervention, compared to the absence in the control supermarkets, will lead to lower perceived effort of purchasing meat substitutes;

HA3 The effect of the social norm intervention, compared to the control supermarkets, on meat substitute purchases is mediated by perceived a) salience and e) effort.

A2. Method

A2.1 Measurement

Items to measure perceived effort and salience are outlined in Table A1. Perceived effort to purchase meat substitutes and perceived salience of meat substitutes were inspired by Raghoebar and colleagues (2020; 2021). All items were measured on a 7-point Likert scale ranging from 1 (Totally disagree) to 7 (Totally agree).

| **Table A1**  *Variable Items for Perceived Effort and Perceived Salience of (Purchasing) Meat Substitutes* | |
| --- | --- |
| Perceived salience | **Item 1** Veggie* products stand out in this [name supermarket]  **Item 2** Veggie products attract attention in this [name supermarket] |
| Perceived effort | **Item 1** Veggie products are easy to find in this [name supermarket]  **Item 2** Veggie products are easy to purchase in this [name supermarket] |
| Note. *“Veggie” in this case translates to “vega” in Dutch, which is a common abbreviation for “vegetarian” or “meat substitute”. “Veggie” (“vega” in Dutch) was explained in the survey. | |

A2.2 Analyses

The two-item variables of perceived effort to purchase meat substitutes and perceived salience of meat substitutes were tested for internal consistency using the Spearman-Brown coefficient (Eisinga, et al., 2013): perceived effort (Spearman-Brown=.866), and perceived salience (Spearman-Brown=.858).

Two separate linear regressions were performed to test the intervention effect on perceived salience(HA1) and perceived effort (HA2). To check whether the conditions for mediation analysis were met (HA3), separate linear regressions were conducted to examine the intervention effect on each perceived salience (HA3a) and perceived effort (HA3b). Additionally, separate linear regressions were performed to assess the effect of perceived salience and effort on the primary outcome variable (i.e., meat substitute purchases). To deal with the zero-inflated outcome variable of meat substitutes, a Two-Part model was used with the *glmmTMB* package in R, in which the first part of the model describes the probability of meat substitute purchases being non-zero and the second part of the model describes the magnitude of the non-zero values (i.e., the amount of meat substitutes purchased in grams among the participant that purchased meat substitutes; Liu et al., 2019; Van der Vliet et al., 2024). If perceived salience and effort showed a significant effect in both pathways, then they were included in the causal mediation analysis. Percentile bootstrapping, generating 95% confidence intervals for indirect effects based on 5000 bootstrap resamples, was applied in the causal mediation analysis.

A3. Results

The average perceived effort to purchase meat substitutes was M=4.7 (SD=1.6) for the total sample, M=4.7(SD=1.6) in the control group, and M=4.7(SD=1.6) in the intervention group. There was no significant difference in perceived effort to purchase meat substitutes between the intervention and the control group (F(1,637)=0.0, p=.972, η^2^=.000). The average perceived salience of meat substitutes was M=4.2 (SD=1.6) for the total sample, M=4.1(SD=1.6) in the control group, and M=4.2(SD=1.7) in the intervention group. There was no significant difference in perceived effort to purchase meat substitutes between the intervention and the control group (F(1,637)=0.0, p=.758, η^2^=.000).

The intervention did not affect the perceived salience of meat substitutes (*β*=0.01, *SE*=0.13, *t*=0.309, *p*=.758), and perceived effort to purchase meat substitutes (*β*=0.00, *SE*=0.13, *t*=0.035, *p*=.972). Perceived salience of meat substitutes and perceived effort to purchase meat substitutes did not affect the likelihood or the amount of meat substitute purchases (Table A2).

| **Table A2**  *Two-Part Models for Effect of Perceived Effort and Perceived Salience on Meat Substitute Purchases.* | | | | |
| --- | --- | --- | --- | --- |
|  | *B* | *SE* | *z* | *p* |
| Perceived effort |  |  |  |  |
| *Part I* | 0.285 | 0.151 | 1.893 | .058 |
| *Part II* | -0.008 | 0.013 | -0.631 | .528 |
| Perceived salience |  |  |  |  |
| *Part I* | 0.103 | 0.130 | 0.794 | .427 |
| *Part II* | -0.001 | 0.013 | -0.040 | .968 |

4. Conclusion

Perceived effort of purchasing meat substitutes and perceived salience of meat substitutes were ruled out as alternative explanations for an intervention effect.
